# Usefulness of multiecho fast field echo MRI in the evaluation of ossification of the posterior longitudinal ligament and dural ossification of the cervical spine

**DOI:** 10.1371/journal.pone.0183744

**Published:** 2017-08-25

**Authors:** So-Yeon Lee, Yu Ri Shin, Hee Jin Park, Myung Ho Rho, Eun Chul Chung

**Affiliations:** 1 Department of Radiology, Kangbuk Samsung Hospital, Sungkyunkwan University School of Medicine, Jongno-gu, Seoul, Republic of Korea; 2 Department of Radiology, College of Medicine, Incheon St. Mary's Hospital, The Catholic University of Korea, Bupyeong-gu, Incheon, Republic of Korea; University of Virginia, UNITED STATES

## Abstract

**Objectives:**

The diagnosis of ossification of the posterior longitudinal ligament (OPLL) on magnetic resonance imaging (MRI) is challenging. The purpose of this study is to evaluate the usefulness of the multiecho fast field echo (mFFE) MRI in the detection of ossification of the posterior longitudinal ligament and dural ossification (DO) of the cervical spine.

**Methods:**

Sixty-three patients who underwent MRI with mFFE and CT for cervical spine were retrospectively evaluated. The presence of OPLL and DO on MR images was assessed by two independent readers. The sensitivity, specificity, and accuracy of MRI for detecting OPLL and DO were determined using CT as a reference standard. Image contrast ratios were obtained between the OPLL and perilesional structures on each sequence.

**Results:**

There were 31 patients with OPLL and 13 DO lesions. The mean sensitivity, specificity, and accuracy of both readers were 94%, 81%, 88% for OPLL and 92%, 81%, 86% for DO, respectively. The contrast ratios for OPLL and intervertebral disc, spinal cord and cerebrospinal fluid were significantly superior on mFFE images, whereas those for OPLL and bone marrow were significantly inferior on mFFE images than those of T1-and T2-weighted images (p ≤ 0.016).

**Conclusions:**

MRI with mFFE may be sufficient for the assessment of OPLL and DO, with good contrasts between OPLL and intervertebral disc, spinal cord, and cerebrospinal fluid.

## Introduction

Ossification of the posterior longitudinal ligament (OPLL) is one of the most important causes of cervical spinal canal stenosis in East Asians. In the diagnosis of cervical spondylotic myelopathy, plain radiography and MRI have been established as the diagnostic methods of choice. However, neither radiography nor conventional magnetic resonance imaging (MRI) is efficient in detecting OPLL. Therefore, computed tomography (CT) is sometimes added to detect OPLL in Asian patients who underwent MRI, since CT is the gold standard in the diagnosis of OPLL [1]. If OPLL and associated findings are evaluated accurately on MRI, patients can save costs and escape exposure to the radiation.

Diagnosis of OPLL is important in patients who are preparing to undergo surgical decompression. Ossified ligaments sometimes tightly adhere to the dura. Surgical removal of OPLL via an anterior approach can result in dural defects and possible cerebrospinal fluid (CSF) leakage [2,3]. Prediction of CSF leakage according to dural ossification (DO) type was suggested by Min and colleagues [3]. In their study, 20 (52.6%) of 38 patients with double-layer signs and three (13.6%) of 22 patients with single-layer signs had surgically confirmed dural defect.

Ossification sites in OPLL are composed largely of lamellar bone with mature Haversian canals. Because gradient-echo magnetic resonance (MR) sequences are helpful for differentiating soft tissue from bony alterations, we made a hypothesis that multiecho fast field echo (mFFE) is useful for detecting OPLL with sufficient contrast to adjacent structure [4]. MFFE is a relatively advanced gradient-echo sequence, which combines multiple bipolar gradient-echo formations. This sequence uses early echoes to increase the signal-to-noise ratio of the image and later echoes to increase image contrast, yielding excellent bone-CSF-soft tissue contrast as well as reducing pulsation artifacts associated with T2-weighted fast spine echo [5,6] This sequence is known by a variety of names, including multiple-echo recombined gradient echo (MERGE) and multiecho data image combination (MEDIC).

The purpose of this study was to evaluate the diagnostic performances of MRI with mFFE, for detecting OPLL and DO. To the best of our knowledge, this is the first report on the diagnostic performance of mFFE for DO as well as OPLL.

## Materials and methods

The study was in compliance with HIPAA guidelines. It was approved by the institutional review board of our institution. The requirement for informed consents was waived by the institutional review board of Kangbuk Samsung Hospital for this retrospective study.

### Study group

Inclusion criteria of this study were as follows: (1) adult, aged >19 years (2) patient who underwent MRI of the cervical spine between January 2012 and June 2013 in our institution (3) patient who underwent CT of the cervical spine between January 2011 and June 2013 in our institution. Exclusion criteria of this study were as follows: (1) Patient who had previously undergone cervical spinal surgery (2) More than two years interval between CT and MRI. Seventy-seven patients met inclusion criteria. Among them Eleven patients were excluded because they had previously undergone cervical spinal surgery. Three patients were excluded because they exhibited severe motion artifacts on their MRI results. A total of 63 patients (36 men, 27 women; mean age, 51 years; range, 20–73 years) were finally included in this study. The mean interval time between CT and MRI was 3.0 months (range, 0–21.6 months).

### CT examination

A 64-channel CT scanner (Brilliance 64; Philips Medical Systems, Best, The Netherlands) was used to obtain 2 mm slices on the sagittal and axial axes. To serve as reference standards, the non-enhanced CT findings of the cervical spine were retrospectively reviewed to consensus by two experienced radiologists [S.Y. L. (reader 1) and H. J. P. (reader 2)]), with 5 and 12 years of experience in musculoskeletal imaging, respectively. When a consensus could not be reached, a third reader with 19 years of experience in spine imaging (M.H.R.) made the final decision. For further evaluation, the cervical spine was divided into C2, C3, C4, C5, C6, and C7 vertebral body levels and C2-3, C3-4, C4-5, C5-6, C6-7, and C7-T1 disc levels. For each segment, the presence of OPLL were evaluated by correlating the axial and sagittal images. The presence and type of DO were also assessed in each patient. If a patient exhibited more than two separate DOs, the data for each DO type were collected separately. All images were evaluated on the bone window (window width/level, 1600–2000 HU / 200–400 HU) on a picture archiving and communication system [7]. The OPLL types were classified as continuous, segmental, mixed, and circumscribed [8]. Continuous type OPLL was defined as a long OPLL extending over several vertebral bodies; segmental type, one or several separate lesions behind the vertebral bodies; mixed type, a combination of continuous and segmental types; and circumscribed type, OPLL mainly located in the disc space. The DO types were classified as isolated, double-layer, and single-layer [9]. Isolated-type DO was defined as ossification of the dura mater without correlation with OPLL; double-layer-type DO was defined as the presence of a non-ossified area between the anterior and posterior ossified rims; and single-layer-type DO was defined as a large focal mass of hyperdense OPLL.

### MRI examination

All MRI examinations were performed using a 1.5-T magnet (Intera; Philips Medical Systems, Best, The Netherlands) using a syn-head coil (Philips Healthcare, 18 coil elements and 8 channels) T1WI (TR range/TE range, 400-700/10-12 msec; turbo factor, 7), T2WI (TR range/ TE range, 2500-3800/100-120 msec; turbo factor, 20–23) and multiecho fast field echo (mFFE) sequence images (TR range/ TE range, 370-460/6-9 msec; flip angle, 25) were obtained in the axial (FOV, 17 cm; matrix, 128–256 × 256, slice thickness, 3 mm; interslice gap, 0.3 mm; signal average 3–6) and sagittal planes (FOV, 24 cm; matrix, 256–512 × 512, slice thickness, 4 mm; no interslice gap; signal average 3). The voxel is anisotropic and each pixel of the plane has a dimension of 0.66–1.30mm for axial images and 0.46–0.93 mm for sagittal images. Each scan time of axial T1WI, T2WI and mFFE, and sagittal axial T1WI, T2WI and mFFE were 2.8 min, 2.8 min, 3.0 min, 2.7 min, 2.7 min, 3.0 min respectively.

MR images were retrospectively and independently analyzed by two readers who were blinded to the CT findings and clinical information (reader 1 and reader 2). To avoid recall bias, MR images were reviewed at least six weeks after review of the CT images. The anatomy of the cervical spine was divided as above, and the presence of OPLL in each segment was scored on a five-point confidence scale: 0 = definitely absent, 1 = probably absent, 2 = equivocal, 3 = probably present, 4 = definitely present. Scores of 0–1 were considered negative, and scores of 2–4 were considered positive [10]. When OPLL scores from 2–4 were obtained, the presence of DO in each patient was scored on a five-point confidence scale, and the type of DO was assessed according to a previously reported classification scheme [9]. The types of DO were recorded separately for patients in whom more than two separate DOs were observed. An overall confidence score, based on all MRI series, was obtained by each reader. Interpretation of the individual sequences was performed without correlating them with any of the other imaging sequences. The suggested diagnostic criteria for OPLL and DO were as follow: OPLL, focal thickening with distinct signal intensity in the posterior longitudinal ligament, distinguishable from perilesional structures; single-layer-type DO, en-bloc thickening of the posterior longitudinal ligament with a meningeal tail; double-layer-type DO, thickening of the anterior and posterior rims of the posterior longitudinal ligament separated by non-ossified posterior longitudinal ligament; isolated DO, focal thickening of the dura mater with normal posterior longitudinal ligament at the same level [9].

The signal intensity in each patient with OPLL was compared with those of the intervertebral disc and muscle intensities in each sequence and interpreted as hyperintense, isointense, hypointense, or mixed signal intensity. Image contrast ratios (CRs) were calculated between the OPLL region and the bone marrow, intervertebral disc, CSF, and cervical spinal cord for the sagittal images on T1WIs, T2WIs, and mFFE images. Polygonal ROIs (size range, 25–45 mm^2^) were drawn in each of the tissues by one radiologist (reader 2), avoiding partial volume artifacts; the resultant signal intensities were then measured. Each CR was calculated as follows: (SI_tissue 1_ –SI_tissue 2_) / (SI_tissue 1_ + SI_tissue 2_), where SI_tissue 1_ is the mean SI of the OPLL, and SI_tissue 2_ is the mean SI of the tissue of interest including bone marrow, intervertebral disc, CSF, and cervical spinal cord.

### Statistical analysis

The diagnostic performance of each sequence was assessed on both a per-person and a per-lesion basis. To determine the diagnostic performance of each sequence, receiver operating characteristic curves were obtained, and the areas under the curves (AUC) were calculated using 95% confidence intervals. The resultant AUCs were then compared with a univariate z test. Interobserver agreement regarding the presence of OPLL and DO in MR images was calculated using weighted κ statistics with agreements of 0.01–0.20, 0.21–0.40, 0.41–0.60, 0.61–0.80, and 0.81–0.99 defined as slight, fair, moderate, substantial, and almost perfect, respectively [11]. The sensitivity, specificity, and accuracy of each MR sequence, including the associated 95% confidence intervals, were also calculated. These values were compared in each sequence using the Cochran Q and McNemar tests. The differences in CRs among the three sequences were evaluated using Friedman’s two-way analysis of variance by ranks. P values were adjusted when multiple comparison were done. A *p* value less than 0.05 was considered statistically significant. All statistical analyses were performed using commercial software (SPSS, version 18, SPSS, Chicago, IL, USA and MedCalc Software, version 11.3.0.0, Mariakerke, Belgium).

## Results

**Fig 1:**
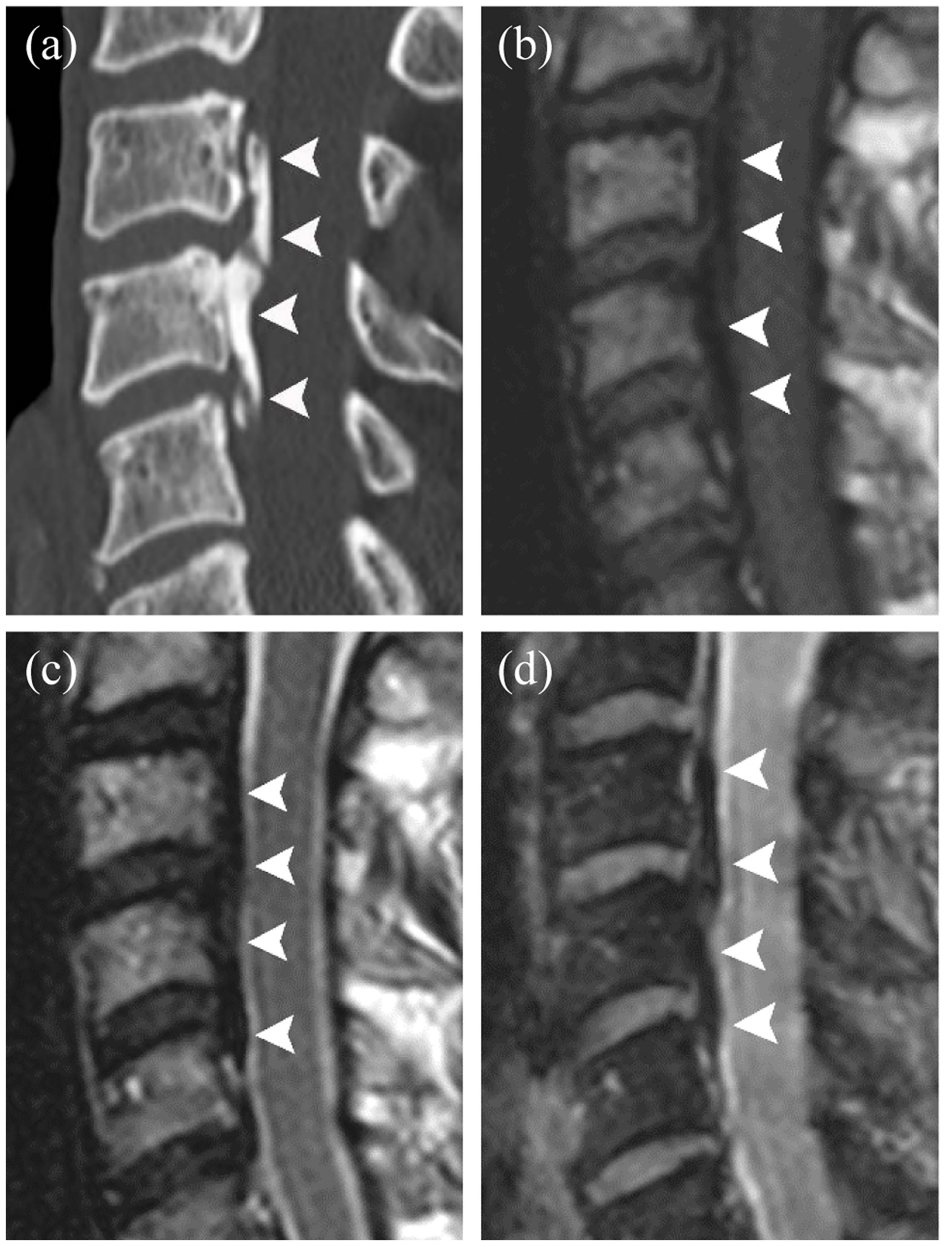
A 56-year-old man with cervical OPLL. **(a)** Sagittal CT of the cervical spine, showing continuous OPLL (*arrowheads*) at the level of the C3 through C5 bodies. **(b)** Sagittal T1WI, showing that the signal intensity of OPLL (*arrowheads*) is similar to that of the CSF. One reader interpreted these findings as an equivocal finding for OPLL, whereas another reader interpreted them as a probable absence of OPLL. **(c)** On the sagittal T2WI, the region of OPLL (*arrowheads*) is not clearly delineated, since its signal intensity is similar to that of the disc. Both readers interpreted these findings as an equivocal finding for OPLL (score 2). **(d)** mFFE image clearly showing a region of OPLL (*arrowheads*) exhibiting good contrast with the CSF and disc. This image was correctly interpreted as indicative of the presence of OPLL (score 4) by both readers.

**Fig 2:**
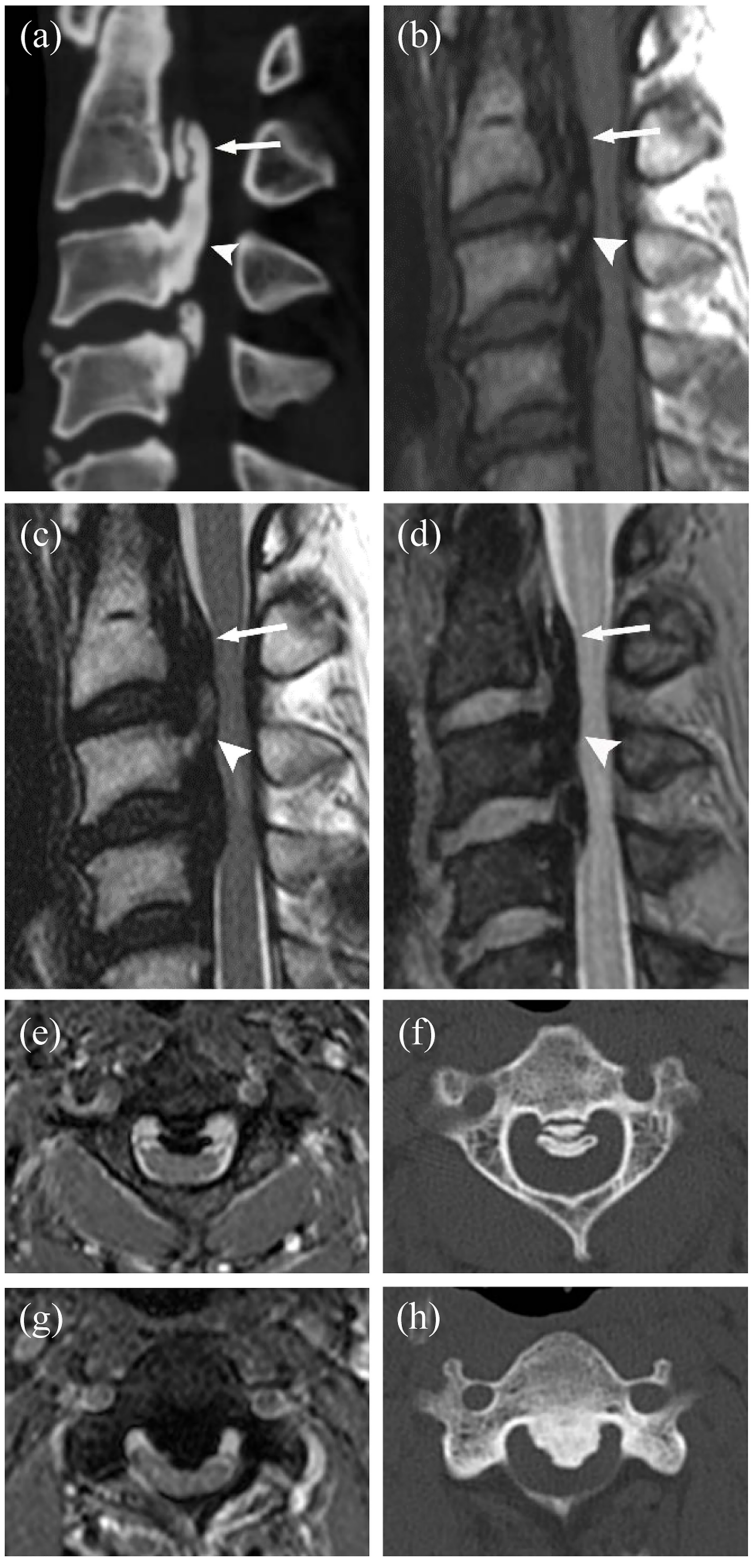
A 42-year-old man with cervical OPLL and DO. **(a)** Sagittal CT image of the cervical spine, showing OPLL with a double-layer DO (arrow) at the C2 body and a single-layer DO (arrowhead) at the C2-3 disc level through the C4 body level. **(b)** Sagittal T1WI showing various signals of OPLL. DO at the C2 level (arrow) was correctly diagnosed by both readers; however, one reader incorrectly interpreted this signal as a single-layer sign. The DO of the C2-3 disc level through C4 body level (arrowhead) was correctly interpreted as a single-layer sign by both readers. **(c)** Sagittal T2WI showing various signals of OPLL. The presence of DO at the C2 level (arrow) was correctly diagnosed by both readers; however, both incorrectly interpreted this signal as a single-layer sign. The DO of the C2-3 disc level through C4 body level (arrowhead) was correctly interpreted as a single-layer sign by both readers. **(d)** Sagittal mFFE image showing a double-layer sign (arrow) and a single-layer sign (arrowhead), which were correctly interpreted as signs of a double-layer and a single layer DO, respectively, by both readers. **(e, f)** Axial mFFE (E) and axial CT (F) images at the C2 body level (arrow, a-d) showing a double layer sign. **(g, h)** Axial mFFE (G) and axial CT (H) images obtained at the C3 body level (arrowhead, a-d) showing a single layer sign.

Of the 63 patients, 31 (23 males, 8 females; mean age, 55 years; range, 42–71 years) had OPLL and 32 (13 males, 19 females; mean age, 47 years; range, 20–73 years) did not. Of the 32 patients without OPLL, 25 had a disc abnormality, five had neural foraminal stenoses, three had bone tumors, one had a fracture of the spinous process, and two patients had no visible abnormalities on their imaging scans, even though each patient experienced neck pain. The types of OPLL observed in the patients included continuous (*n* = 10), segmental (*n* = 9), mixed (*n* = 9), and circumscribed (*n* = 3) (Fig 1). Of the 31 patients with OPLL, 13 (42%) had DO, including one with isolated type, 12 with double-layer type and eight with single-layer type (Fig 2). Eight patients had two separate DO lesions each. The signal intensity of OPLL varied on the T1WIs (18 hypointense, 8 hyperintense, 4 mixed, and 1 isointense) and on the T2WIs (15 hypointense, 7 hyperintense, 7 mixed, and 2 isointense) but not on the mFFE images (all hypointense).

The AUCs, sensitivities, specificities, and accuracies of all MR sequences for the detection of OPLL were in Table 1. The AUCs, sensitivities, specificities, and accuracies of all MR sequences for the detection of DO were in Table 2. The AUCs of mFFE in per-person analysis for the diagnosis of OPLL were significantly greater than those of T1WI and T2WI on sagittal images (*P* = 0.001–0.028) but not significantly different on axial images (*p* = 0.116–0.135) (Tables 3 and 4). As for per-lesion analysis, the AUCs, sensitivities, specificities, and accuracies of all MR sequences for the detection of OPLL were in Table 5. The AUCs of mFFE the diagnosis of OPLL were greater than those of T1WI and T2WI, on both sagittal and axial images at both the vertebral body and disc levels (*p* ≤ 0.045). The sensitivity and specificity of sagittal mFFE the diagnosis of OPLL were superior to those of T1WI and T2WI, at both the vertebral body and disc levels (*p* ≤ 0.022). Regarding comparisons between sagittal and axial images and vertebral body and disc levels, no significant differences were observed in the AUCs for detection of OPLL (*p* = 0.422–0.946). Interobserver agreements for the diagnosis of OPLL were classified as almost perfect (κ = 0.833–0.983).

**Table 1:** Overall diagnostic performance of MRI in the diagnosis of OPLL.

Statistics	OPLL
AUC	
reader 1	0.938 (0.847–0983)
reader 2	0.961 (0.879–0.993)
Sensitivity (%)	
reader 1	90.3 [28/31] (79.3–1.00)
reader 2	96.8 [30/31] (90.2–1.00)
Specificity (%)	
reader 1	81.3 [26/32] (67.0–95.6)
reader 2	81.3 [26/32] (67.0–95.6)
Accuracy (%)	
reader 1	85.7 [54/63] (76.8–94.6)
reader 2	88.9 [56/63] (80.9–96.9)

Abbreviations: OPLL, ossification of posterior longitudinal ligament; AUC, area under the receiver operating characteristic curve.

Numbers in parentheses represent 95% confidence intervals and data in brackets represent the numbers of lesions.

**Table 2:** Overall diagnostic performance of MRI in the diagnosis of DO.

Statistics	DO
AUC	
reader 1	0.912 (0.754–0.984)
reader 2	0.908 (0.749–0.982)
Sensitivity (%)	
reader 1	92.3 [12/13] (75.6–1.00)
reader 2	92.3 [12/13] (75.6–1.00)
Specificity (%)	
reader 1	83.3 [15/18] (64.3–1.00)
reader 2	77.8 [14/18] (56.5–99.1)
Accuracy (%)	
reader 1	87.1 [27/31] (74.6–99.6)
reader 2	83.9 [26/31] (70.2–97.6)

Abbreviations: DO, dural ossification; AUC, area under the receiver operating characteristic curve.

Numbers in parentheses represent 95% confidence intervals and data in brackets represent the numbers of lesions.

**Table 3:** Overall diagnostic performance of MRI in the diagnosis of OPLL and per-lesion analysis at vertebral body levels.

Statistics	Vertebral body level
AUC	
reader 1	0.960 (0.935–0.978)
reader 2	0.961 (0.937–0.978)
Sensitivity (%)	
reader 1	83.2 [84/101] (75.8–90.6)
reader 2	82.2 [83/101] (74.6–89.8)
Specificity (%)	
reader 1	98.6 [273/277] (97.1–1.00)
reader 2	98.9 [274/277] (97.7–1.00)
Accuracy (%)	
reader 1	94.4 [357/378] (92.1–96.8)
reader 2	94.4 [357/378] (92.1–96.8)

Abbreviations: OPLL, ossification of posterior longitudinal ligament; AUC, area under the receiver operating characteristic curve.

Numbers in parentheses represent 95% confidence intervals and data in brackets represent the numbers of lesions.

**Table 4:** Overall diagnostic performance of MRI in the diagnosis of OPLL and per-lesion analysis at disc levels.

Statistics	Disc level
AUC	
reader 1	0.974 (0.952–0.987)
reader 2	0.972 (0.950–0.986)
Sensitivity (%)	
reader 1	89.7 [52/58] (81.6–97.7)
reader 2	89.7 [52/58] (81.6–97.7)
Specificity (%)	
reader 1	93.4 [299/320] (90.7–96.2)
reader 2	91.6 [293/320] (88.5–94.6)
Accuracy (%)	
reader 1	92.9 [357/378] (90.3–95.5)
reader 2	91.3 [345/378] (88.4–94.1)

Abbreviations: OPLL, ossification of posterior longitudinal ligament; AUC, area under the receiver operating characteristic curve.

Numbers in parentheses represent 95% confidence intervals and data in brackets represent the numbers of lesions.

**Table 5:** Diagnostic performance of each MR sequence in the diagnosis of OPLL.

MRI Axis	AUC			Comparison (*P* value)	
	T2WI	T1WI	mFFE	mFFE vs. T2WI	mFFE vs. T1WI
Sagittal					
reader 1	0.809 (0.715–0.904)	0.848 (0.763–0.933)	0.940 (0.890–0.990)	0.001*	0.012*
reader 2	0.873 (0.765–0.944)	0.895 (0.792–0.958)	0.953 (0.867–0.990)	0.012*	0.028*
Axial					
reader 1	0.902 (0.841–0.964)	0.904 (0.835–0.973)	0.934 (0.880–0.989)	0.135	0.116
reader 2	0.927 (0.833–0.978)	0.925 (0.831–0.976)	0.955 (0.870–0.991)	0.118	0.121

Abbreviations: OPLL, ossification of posterior longitudinal ligament; AUC, area under the receiver operating characteristic curve; T2WI, T2-weigted MR imaging; T1WI, T1-weigted MR imaging; mFFE, multiecho fast field echo.

Numbers in parentheses represent 95% confidence intervals.

*, statistically significant (P < .050)

The AUCs of mFFE for the detection of DO for both readers were significantly greater than those of T1WI and T2WI on sagittal images (*p* = 0.001–0.010) but were not significantly different on axial images (*p* = 0.162–0.320) in per-person analysis (Table 6). Regarding per-lesion analysis on all MR sequences, 83.3% (10/12) of all double-layer type lesions were correctly diagnosed as DO by both readers, 80% (8/10) of all single-layer type lesions were correctly diagnosed as DO by both readers, but none (0/1) of the isolated type lesions were correctly diagnosed as DO by either reader. Most lesion types were correctly classified; however, a few double-layer lesions (*n* = 3 for reader 1 and *n* = 4 for reader 2) were misclassified as single-layer-type. Interobserver agreements for the diagnosis of DO ranged from substantial to almost perfect (κ = 0.867–0.963).

**Table 6:** Diagnostic performance of each MR sequence in the diagnosis of DO.

MRI Axis	AUC			Comparison (*P* value)	
	T2WI	T1WI	mFFE	mFFE vs. T2WI	mFFE vs. T1WI
Sagittal					
reader 1	0.741 (0.553–0.881)	0.703 (0.513–0.853)	0.947 (0.801–0.995)	0.002*	0.001*
reader 2	0.769 (0.584–0.901)	0.744 (0.556–0.883)	0.919 (0.766–0.986)	0.009*	0.010*
Axial					
reader 1	0.748 (0.560–0.886)	0.759 (0.572–0.893)	0.803 (0.622–0.924)	0.320	0.173
reader 2	0.816 (0.636–0.932)	0.821 (0.641–0.934)	0.868 (0.697–0.962)	0.304	0.162

Abbreviations: OPLL, ossification of posterior longitudinal ligament; AUC, area under the receiver operating characteristic curve; T2WI, T2-weigted MR imaging; T1WI, T1-weigted MR imaging; mFFE, multiecho fast field echo.

Numbers in parentheses represent 95% confidence intervals.

*, statistically significant (P < .050)

The CRs for OPLL and perilesional structures were significantly different among T2WI, T1WI and mFFE (*P* < 0.001). Post-hoc test revealed that the CRs for OPLL-disc, OPLL-cord and OPLL-CSF were significantly superior on mFFE images in comparison to T1WI or T2WI scans (*p* ≤ 0.016), whereas those for OPLL-BM were significantly inferior on mFFE images in comparison to T1WI and T2WI scans (*p* < 0.001) (Table 7).

**Table 7:** Image contrast ratios of each MR sequence.

Category	Contrast Ratio			Comparison (*P* value)	
	T2WI	T1WI	mFFE	mFFE vs. T2WI	mFFE vs. T1WI
OPLL-disc	0.37 (0.20–0.55)	0.42 (0.16–0.65)	0.62 (0.55–0.69)	< 0.001*	0.001*
OPLL-cord	0.51 (0.34–0.72)	0.44 (0.17–0.64)	0.68 (0.62–0.75)	0.016*	< 0.001*
OPLL-CSF	0.75 (0.67–0.90)^c^	0.38 (0.28–0.51)	0.78 (0.74–0.83)	1.000	< 0.001*
OPLL-BM	0.61 (0.48–0.79)^d^	0.57 (0.37–0.76)	0.32 (0.18–0.43)	< 0.001*	< 0.001*

Abbreviations: T2WI, T2-weigted MR imaging; T1WI, T1-weigted MR imaging; mFFE, multiecho fast field echo; OPLL, ossification of posterior longitudinal ligament; CSF, cerebrospinal fluid; BM, bone marrow.

Numbers in parentheses represent 95% confidence intervals.

*, statistically significant (*P* < .050)

## Discussion

We found that mFFE sequences were highly accurate in diagnosing the presence of OPLL and DO. In contrast OPLLs were not clearly distinguished from normal tissue on T1WI and T2WI sequences probably because of variations in OPLL signals [12]. Using these sequences, the diagnosis of OPLL is dependent on the thickening and shape of the posterior longitudinal ligament, therefore differentiation from disc herniation is sometimes difficult.

In previous study by Otake et al. [4], the sensitivity of MRI was greater in axial images [74% (20/27) on T1WI, 98% (44/45) on intermediate-weighted images, 91% (41/45) on T2WI, 89% (16/18) on fast low angle shot (FLASH) images, and 93% (79/85) on fast imaging with steady-state precession (FISP)] than in sagittal images [33% (48/147) on T1WI, 70% (92/131) on intermediate-weighted images, and 44% (58/131) on T2WI], using lateral tomography as a reference standard. Similarly to Otake et al’s study, we also found the relatively superior diagnostic performance of axial imaging compared to sagittal imaging on T1- and T2-weighted imaging. However, the diagnostic performance of sagittal mFFE images was as superior as that of axial mFFE images in our study.

To the best of our knowledge, only one report to date has studied the diagnostic performance of MRI for the detection of DO. In that report, 12/17 patients with OPLL were correctly diagnosed using T2WI scans, compared with 0/17 patients with DO [9]. We found superior diagnostic performance of MRI with mFFE for the detection of DO as compared with T1WI and T2WI.

The rate of OPLL was higher in our study population than expected for East Asians [8]. Tsuyama reported a 2.4% incidence of OPLL in Asian populations and a 0.16% incidence in non-Asian populations. Several factors may increase this incidence. Previous reports of the prevalence of OPLL were based on radiography in healthy populations. Our study is based on patients who have cervical pathology as severe as considering special imaging. We also included tiny OPLL because the reference standard was not radiography, but CT. In addition, our hospital is tertiary hospital where many patients are referred for surgery, which could have led to selection bias.

This study had several limitations, including its retrospective design. The retrospective nature of this study resulted in CT and MRI scans at different time points and potentially biased the final sample. We found that MRI with mFFE performed well in diagnosing DO as well as OPLL, with the added benefit that radiation exposure could be avoided. However, it is still unknown whether MRI can replace CT, either in part or in whole. To fully answer this question, future prospective studies with larger case numbers are required. Our study may have also been subject to substantial selection bias due to its exclusion of patients for whom CT was not performed. More severe pathology subtypes and therefore more obvious OPLL and DO were included; this might have biased the detection rates for MRI. Moreover, for the sagittal images, 4-mm-thick slices were used, which may have resulted in only partial volumes being observed and thus have led to false negative findings regarding small calcifications. The incidence of OPLL is relatively higher than the normal population, indicating that diagnostic performance can vary. We were unable to compare the diagnostic performances of mFFE with intermediate-weighted imaging, or of FLASH with FISP, because these procedures were not included in the routine MRI protocol of our hospital. Another weakness of our study was that the number of patients with DO was relatively small. Furthermore, a significant proportion of the double-layer signs in this study were interpreted as single-layer signs, which may have resulted from susceptibility artifacts on gradient-echo images. The double-layer sign has been shown to have a significant association with dural penetration, whereas the clinical meaning of the single-layer sign remains unclear. We obtained and compared CR, but which can be affected by noise. Finally, some patients exhibited long time intervals between CT and MRI.

## Conclusion

MRI with mFFE may be sufficient for the assessment of OPLL and DO, but that CT should still be considered for cases in which questions remain.
